# Systematic review protocol: evaluation of candidate platforms and vaccines for emerging and re-emerging viral threats

**DOI:** 10.1186/s13643-025-03051-3

**Published:** 2026-01-10

**Authors:** Amit Bansal, Ida Sofie Karlsen Sletten, Tung Thanh Le, Stig Tollefsen, John P. Shannon, Rebecca Jane Cox, Rishi Delan Pathirana, UiB-CEPI Group, Malik Beglerovic, Bjørn Blomberg, Karl Albert Brokstad, Elin Hoffmann Dahl, Kurt Hanevik, Tuva Småland Hagland, Thorkild Tylleskär

**Affiliations:** 1 Department of Clinical Science, Influenza Centre, University of Bergen, Bergen, Norway; 2 Medical Library, University of Bergen, Bergen, Norway; 3 Coalition for Epidemic Preparedness Innovations (CEPI), Oslo, Norway; 4 Norwegian Institute of Public Health, Oslo, Norway

**Keywords:** Coronavirus, Lassa fever, Nipah virus, Mpox, Emerging infectious diseases, Vaccines, Viral vector vaccines, Protein subunit vaccines, RNA vaccines, Mucosal immunity

## Abstract

**Background:**

Emerging viral threats such as coronavirus, influenza, Lassa fever, Mpox, and Nipah virus continue to pose significant global health challenges. The development and deployment of effective vaccines are essential for outbreak control and pandemic preparedness. This protocol describes a systematic review that will synthesize evidence on vaccine candidates targeting high-priority viral threats and major vaccine platforms.

**Methods:**

We will include randomised controlled trials (RCTs) assessing vaccine candidates for specified viruses (coronavirus, Lassa fever, Nipah virus, and Mpox) and platforms (protein-based, viral vector, and RNA) in the human populations. To encompass the full vaccine development landscape, exploratory and preclinical studies may also be included (any type of original research). Data sources will include MEDLINE Ovid, Embase Ovid (including ClinicalTrials.gov), and Cochrane Library (including CENTRAL), and grey literature (including conference proceedings, dissertations, trial registries, and company websites). Risk of bias will be assessed using Cochrane revised tool for assessing risk of bias in randomised trials (RoB-2) and Systematic Review Centre for Laboratory Animal Experimentation (SYRCLE), and certainty of evidence will be evaluated using Grading of Recommendations Assessment, Development and Evaluation (GRADE). The primary outcomes are safety, immunogenicity, and vaccine efficacy or effectiveness, prioritised for their critical role in outbreak management and in guiding regulatory approval, public health policy, and clinical decision-making. Meta-analyses will be conducted where appropriate, using both fixed- and random-effects models. Subgroup analyses will be performed to explore heterogeneity based on virus type, vaccine platform, and outcome measures, where appropriate.

**Discussion:**

This review will provide a comprehensive synthesis of vaccine development efforts across multiple platforms and pathogens with epidemic or pandemic potential. It will inform future research, policy, and investment decisions in global health preparedness.

**Systematic review registration:**

PROSPERO 2025 CRD420251082338 on 6 October 2025.

**Supplementary Information:**

The online version contains supplementary material available at 10.1186/s13643-025-03051-3.

## Background

The global burden of emerging viral diseases has intensified in recent decades, with outbreaks of coronavirus, Nipah virus, Lassa virus, and Mpox highlighting the urgent need for scalable and effective vaccine platforms [1–3]. Traditional vaccine development pathways are often slow and resource-intensive, prompting a shift towards platform-based approaches that can be rapidly adapted to new pathogens. Recent advances in RNA technology [4–6] and viral vectors delivery systems have demonstrated promising results in both preclinical and clinical settings [7–10]. The regulatory approval of vaccines is predicated on a rigorous scientific assessment demonstrating that their anticipated benefits outweigh potential risks, compliance with Good Manufacturing Practice (GMP), and evidence of unmet clinical need [11]. Nevertheless, there remains a notable paucity of comprehensive data concerning key vaccine platforms and candidates targeting high-priority viruses with pandemic potential. In particular, substantial gaps persist in the literature regarding manufacturing platforms, technological modalities, delivery mechanisms, affordability, accessibility, cost of goods, geographical distribution, and long-term sustainability. These factors are critical to the global deployment and equity of vaccines and must be considered alongside the expanding evidence base on safety, immunogenicity, and clinical efficacy or real-world effectiveness.

This systematic review aims to map and analyse the landscape of vaccine candidates and platforms, providing insights into their efficacy, safety, immunogenicity, and practical vaccine deployment considerations. The objective is to evaluate the current landscape of vaccine candidates targeting specified viruses (coronavirus, Nipah, Lassa, and Mpox) and technological platforms (protein-based, viral vector, and RNA), across the entire development life cycle, from pre-clinical stages through to commercialisation. Previous reviews have often focused on a single pathogen or platform, whereas these reviews adopt a broader approach to aid in the generation of a new vaccine that can respond rapidly to the next unknown threat, avoiding progression to the pandemic stage.

## Methods

This protocol follows the preferred reporting items for systematic review and meta-analysis protocols (PRISMA-P) 2015 statement [12].

### Eligibility criteria

The inclusion/exclusion criteria for selecting studies will be based on the Coalition for Epidemic Preparedness Innovations (CEPI) objectives to develop vaccines promptly in the event of an outbreak/pandemic [13, 14], which could include the following:

#### Inclusion criteria

To ensure methodological rigour in the assessment of clinical studies, only randomised controlled trials (RCTs) will be included in the human populations, evaluating the efficacy and/or safety of vaccine candidates (coronavirus, Nipah, Lassa, Mpox) and vaccine platforms (protein, viral vector, and RNA). Nevertheless, to capture recent advancements in vaccine technologies, exploratory and preclinical studies will also be considered.

Limits: No language restrictions. Searches will include records published within the two years preceding the final search date, although this timeframe may be extended in specific circumstances, such as when data availability for a particular concept map during that period is limited. The search strategy will be revised in consultation with our teams to meet key objectives.

Studies reporting data on safety outcomes (e.g., adverse events, serious adverse events).

Studies reporting data on clinical efficacy (e.g., prevention of infection, disease severity) or immunogenicity (e.g., antibody titres, T-cell responses).

Studies reporting data on manufacturing platforms, modality, delivery systems, affordability, accessibility, cost of goods, geographical diversity, and sustainability.

#### Exclusion criteria

Observational studies, reviews, editorials, and commentaries.

Non‐randomised and modelling studies in human populations.

Studies not reporting relevant immunogenicity, efficacy, or safety outcomes in human populations.

#### Population, Interventions, Comparators, and Outcomes (PICO) framework

This review will include human participants of any age or health status. Where appropriate, preclinical animal models will also be considered for inclusion in the narrative synthesis to provide contextual insights. The interventions of interest are vaccines targeting coronavirus, influenza, Nipah, Lassa, and Mpox, developed using various platforms including protein-based, RNA, and viral vector technologies. Comparators will include placebo controls or alternative vaccine formulations. The primary outcomes to be assessed are vaccine efficacy or effectiveness, immunogenicity, safety, and durability. Secondary outcomes will include manufacturing metrics, accessibility, and cost of goods.

### Information sources

#### Databases

MEDLINE Ovid, Embase Ovid (including ClinicalTrials.gov), and Cochrane Library including Cochrane Central Register of Controlled Trials (CENTRAL).

#### Grey literature

Manual searches including conference proceedings, dissertations, trial registries, and company websites. For instance, conference proceedings such as Vaccine Congress, International Society for Influenza and other Respiratory Virus Diseases (ISIRV), and European Society of Clinical Microbiology and Infectious Diseases (ESCMID); dissertation platforms such as the Bergen Open Research Archive (BORA) accessible through the University of Bergen; trial registries including ClinicalTrials.gov and the World Health Organization International Clinical Trials Registry Platform (WHO ICTRP). Study authors will be contacted in the event of difficulties accessing articles or when clarification is required.

### Search strategy

A comprehensive search strategy will be developed for each database in collaboration with experienced academic librarian(s). Manual searches and expert consultations will supplement electronic searches. Specific search strategies for a given landscape topic will be developed. Free text synonym searches in title/abstract/keywords fields, and relevant MeSH/Emtree subject headings, will be combined with Boolean operators for broad information retrieval. Truncation and adjacency will be utilised to make the search more inclusive.

Example search algorithms for MEDLINE Ovid, Embase Ovid, and CENTRAL are shown in Supplementary tables 1–3, including protein-based vaccine landscape, viral vector vaccine landscape, and vaccines against Nipah and Lassa fever viruses. A similar strategy will be adapted for selected viruses and platforms to meet this project’s key objectives in consultation with our subject group experts.

### Study records

#### Selection process

Two independent reviewers will perform an initial title and abstract screening using predefined criteria and conduct a full-text review of shortlisted studies. The screening will focus on specific aspects of vaccine landscapes, which can include vaccine platform, safety, efficacy/effectiveness, routes of administration, manufacturing sites, adjuvants, delivery systems, sustainability, and access to the technology (including freedom to operate, intellectual property, thermostability or other parts of access such as sustainability and equity). Any discrepancies in the screening process will be resolved by consensus. A third review author will be called upon to resolve conflict(s), if deemed necessary.

#### Data screening procedure

For screening and full-text review, studies or trial results identified through search algorithms will be exported (in EndNote XML, PubMed text format, or RIS text format), duplicates removed [15] (Research Resource Identifier, RRID:SCR_025607), and subsequently imported to AI-assisted software. Data extraction is traditionally a manual and error-prone process that is increasingly being automated through the use of natural language processing (NLP) and machine learning (ML) algorithms. We will utilise tools such as Rayyan and Covidence; however, we are currently exploring additional platforms—including Scispace, SWIFT–Review, ASReview LAB, and similar AI-assisted tools—to support the systematic review process [16].

#### Data collection

Data will be extracted independently by two reviewers using a piloted form. While extracting data, authors may be contacted to provide more information on missing data or for clarifications, as required.

### Data items

Variables include vaccine type, platform, delivery method, dosage, efficacy outcomes, immunogenicity markers, adverse events, and manufacturing/accessibility indicators.

### Outcomes and prioritisation

This systematic review will seek data on both primary and secondary outcomes, selected to enable a comprehensive assessment of the performance, safety, and broader implications of vaccine candidates [17]. The primary outcomes are: (i) vaccine efficacy or effectiveness, defined as the reduction in disease incidence among vaccinated individuals compared to unvaccinated or active controls (e.g., placebo or standard of care controlled), preferably measured in clinical trials; (ii) immunogenicity, assessed through quantitative indicators such as antibody titres and T-cell responses, which serve as proxies for the immune response elicited by vaccination; and (iii) safety, evaluated through the incidence and severity of adverse events (AEs) and serious adverse events (SAEs), as reported in clinical trials or observational studies. These outcomes are prioritised due to their critical importance in informing regulatory approval, public health policy, and clinical decision-making. The secondary outcomes include: (i) manufacturing feasibility, referring to the technical and logistical capacity to produce the vaccine at scale; (ii) cost of goods, defined as the estimated production cost per dose (including estimated costs of ingredients and running costs), which influences affordability and procurement strategies; (iii) accessibility, understood as the extent to which vaccines are available (including freedom to operate, intellectual property, thermostability), and equitably distributed across populations and regions; and (iv) scalability, referring to the potential for rapid expansion of production and distribution in response to public health demand. These secondary outcomes are included to provide insight into the practical, economic, and implementation-related considerations that affect vaccine deployment and global health impact.

### Risk of bias assessment

Cochrane revised tool for assessing risk of bias in randomised trials (RoB-2) will be used to assess the risk of bias in RCTs. For animal studies, Systematic Review Centre for Laboratory Animal Experimentation (SYRCLE) risk of bias tool will be used. Assessments will be conducted independently by two reviewers.

### Data synthesis

We will extract data on study details, for example, author, year, location, vaccine platform, target virus, trial phase, efficacy, safety, and immunological outcomes using a standardised data extraction form (consolidated database) in consultation with our team members. Data will be eventually stored in a dedicated database for vaccine and platform landscape.

Separate descriptive analyses will be conducted for the human and animal cohorts, and these will be disease- or platform-specific. Animal studies will primarily involve narrative analysis. Meta-regression analyses will be conducted exclusively on human RCTs, subject to the availability of resources and time. A structured approach will be employed to quantitatively synthesize findings from eligible studies:

#### Meta-analysis models and statistical methods

Where appropriate, data will be pooled using fixed-effect and/or random-effects meta-analysis models [18, 19]. Statistical techniques such as Mantel–Haenszel, inverse variance weighting, and DerSimonian–Laird estimators will be selected based on the nature of the data and the underlying assumptions of each method.

#### Assessment of statistical heterogeneity

Heterogeneity across studies will be evaluated through visual inspection of forest plots and the Luis Furuya-Kanamori (LFK) index, formal statistical tests (e.g., Cochran’s Q), and measures of heterogeneity variance (τ^2^) and inconsistency (e.g., I^2^ statistic). These assessments will guide the interpretation of pooled estimates and inform model choice. Statistical heterogeneity among included studies will be assessed using both the Chi-squared (χ^2^) test and the I^2^ statistic. A *p*-value of less than 0.1 in the χ^2^ test will be considered indicative of statistically significant heterogeneity. The I^2^ statistic will be used to quantify the proportion of total variation across studies attributable to heterogeneity rather than chance. An I^2^ value of less than 30% will be interpreted as indicating mild heterogeneity, values between 30 and 50% as moderate heterogeneity, and values exceeding 50% as substantial heterogeneity. In addition, the R^2^ statistic will be employed to quantify the proportion of variability explained by covariates included in the random-effects meta-regression model, where applicable. These measures will guide decisions regarding the appropriateness of meta-analysis and the need for subgroup or sensitivity analyses.

#### Planned subgroup analyses

Subgroup analyses will be conducted to explore potential sources of heterogeneity and enhance interpretability. These may include stratification based on: risk of bias (e.g., comparing high-risk versus low-risk studies), circulating variants, vaccine type, disease outcome type (e.g., infection incidence versus disease severity), virus type, vaccine platform, and route of administration. The following factors will be analysed to delineate their effect on vaccine safety, immunogenicity, and efficacy/effectiveness in human trials: disease (e.g., symptomatic or asymptomatic, severity), virus (strain), vaccine platform, route of administration, vaccine type, and vaccine dosage.

Risk ratios with 95% confidence intervals (CIs) will be calculated for dichotomous outcomes such as analysis of adverse events in human trials. Random- and fixed-effects models will be employed to assess immunogenicity, and to account for the heterogeneity of both humoral and cell-mediated immunogenicity, where appropriate.

### Meta-bias(es) and confidence in cumulative evidence

Potential meta-biases, including publication bias and selective outcome reporting, will be assessed through visual inspection of funnel plots, where appropriate, and quantified using LFK indices. Publication bias will be assessed only when 10 or more studies are included in a systematic review or meta-analysis. These methods will help identify asymmetry suggestive of small-study effects or reporting bias. To evaluate the overall certainty of the evidence, the Grading of Recommendations Assessment, Development and Evaluation (GRADE) approach will be employed [20]. This framework considers factors such as risk of bias, consistency of results, directness of evidence, precision of estimates, and potential publication bias, thereby providing a transparent and structured assessment of the strength of the body of evidence for each outcome.

## Systematic review registration

The protocol was registered in PROSPERO (CRD420251082338) on 6 October 2025 [21].

## Discussion

This protocol outlines a systematic review that will synthesize evidence on vaccine candidates targeting high-priority viral threats and major vaccine platforms. By focusing on RCTs, the review will provide robust insights into comparative effectiveness, safety, and immunogenicity across platforms in the human populations. The inclusion of manufacturing and accessibility metrics will enhance the relevance of findings for policymakers and funders. The evidence may prove valuable to clinicians, patients, and health policymakers in facilitating the timely deployment of vaccines during viral outbreaks. Given the dynamic nature of vaccine and platform development, this review may serve as a foundation for a living database to support global health pandemic preparedness.

## Supplementary Information

Supplementary material 1: PRISMA-P 2015 Checklist.

Supplementary material 2: search algorithms.

## Data Availability

No datasets were generated or analysed during the current study.

## Abbreviations

CEPI: Coalition for Epidemic Preparedness Innovations
CENTRAL: Cochrane Central Register of Controlled Trials
GRADE: Grading of Recommendations Assessment, Development and Evaluation
LFK index: Luis Furuya-Kanamori index
MeSH: Medical Subject Headings
ML: Machine learning
NLP: Natural language processing
PICO: Population, Interventions, Comparators, and Outcomes
PROSPERO: The International Prospective Register of Systematic Reviews
RCTs: Randomised controlled trials
RoB-2: Cochrane revised tool for assessing risk of bias in randomised trials
SYRCLE: Systematic Review Centre for Laboratory Animal Experimentation
UiB: University of Bergen, Bergen, Norway
χ^2^: Chi-squared
