# Supplementary material for: Systematic review protocol: evaluation of candidate platforms and vaccines for emerging and re-emerging viral threats
Source: Syst Rev. 2026 Jan 10;15:49. doi: 10.1186/s13643-025-03051-3 (PMC12882252; doi:10.1186/s13643-025-03051-3)
Supplement: Supplementary file 2 — Supplementary material 2: search algorithms. [file 13643_2025_3051_MOESM2_ESM.docx]

**Supplementary table 1: Literature search algorithms for protein-based vaccine technology landscape using MEDLINE Ovid, Embase Ovid and CENTRAL**

| **MEDLINE(R) Ovid, Date: 14.10. 2025** | | |
| --- | --- | --- |
| 1 | Vaccines, Virus-Like Particle/ or Nanovaccines/ or exp Vaccines, Subunit/ | 11627 |
| 2 | (((protein* or peptide* or recombinant or subunit* or nanoparticle* or virus like particle* or virus-like particle*) adj2 vaccin*) or nanovaccin* or proteinvaccin* or peptidevaccin* or subunitvaccin* or recombinantvaccin*).ti,ab,kf. | 30427 |
| 3 | 1 or 2 | 37248 |
| 4 | exp Viruses/ or exp Virus Diseases/ | 1781627 |
| 5 | (virus or viral).ti,ab,kf. | 1108401 |
| 6 | 4 or 5 | 2069843 |
| 7 | 3 and 6 | 20169 |
| 8 | limit 7 to last 2 years | 3791 |
| 9 | limit 8 to (case reports or meta analysis or network meta-analysis or "review" or "scoping review" or "systematic review") | 663 |
| 10 | 8 not 9 | 3128 |
| 11 | limit 10 to (clinical study or clinical trial, all or clinical trial, phase i or clinical trial, phase ii or clinical trial, phase iii or clinical trial, phase iv or clinical trial, veterinary or clinical trial protocol or clinical trial or controlled clinical trial or human challenge trials or randomized controlled trial or randomized controlled trial, veterinary) | 217 |
| 12 | (rct or ((random* or clinical or preclinical or multicenter) adj3 (study or studies or trial*)) or ((animal* or human* or invitro or in-vitro or in vitro) adj3 (model* or challenge))).ti,ab,kf. | 1944785 |
| 13 | 10 and 12 | 470 |
| **14** | **11 or 13** | **519** |
| Hyperlink: <https://ovidsp.ovid.com/ovidweb.cgi?T=JS&NEWS=N&PAGE=main&SHAREDSEARCHID=5CgyQeq2OKNv6RM32FnQqxPFUgAOZTdzwOYEg0qE1EMOXpsr0HiaV4wfazAI1AngB> | | |
| **Embase Ovid, Date: 14.10. 2025** | | |
| 1 | protein vaccine/ or nanovaccine/ or peptide vaccine/ or recombinant vaccine/ or subunit vaccine/ or virus like agent/ | 27214 |
| 2 | (((protein* or peptide* or recombinant or subunit* or nanoparticle* or virus like particle* or virus-like particle*) adj2 vaccin*) or nanovaccin* or proteinvaccin* or peptidevaccin* or subunitvaccin* or recombinantvaccin*).ti,ab,kf. | 37985 |
| 3 | 1 or 2 | 52672 |
| 4 | exp virus infection/ or exp virus/ | 2966522 |
| 5 | (virus* or viral).ti,ab,kf. | 1390568 |
| 6 | 4 or 5 | 3181515 |
| 7 | 3 and 6 | 32358 |
| 8 | limit 7 to last 2 years | 5371 |
| 9 | limit 8 to (books or chapter or "review") | 882 |
| 10 | limit 8 to (meta analysis or "systematic review") | 110 |
| 11 | 8 not (9 or 10) | 4443 |
| 12 | limit 11 to (clinical trial or randomized controlled trial or controlled clinical trial or multicenter study or phase 1 clinical trial or phase 2 clinical trial or phase 3 clinical trial or phase 4 clinical trial) | 534 |
| 13 | (rct or ((random* or clinical or preclinical or multicenter) adj3 (study or studies or trial*)) or ((animal* or human* or invitro or in-vitro or in vitro) adj3 (model* or challenge))).ti,ab,kf. | 2961762 |
| 14 | 11 and 13 | 689 |
| **15** | **12 or 14** | **902** |
| Hyperlink: <https://ovidsp.ovid.com/ovidweb.cgi?T=JS&NEWS=N&PAGE=main&SHAREDSEARCHID=4umlX5AxfPtner9HKlCqgYa8e4wE4XgQhVfrdVqJx4RQ2AUhqyljHdQQ1KJULDnZN> | | |
| **CENTRAL (Cochrane's database of trials), Date: 14.10.2025** | | |
| #1 | (((protein* OR peptide* OR recombinant OR subunit* OR nanoparticle* OR "virus like" NEXT particle* OR "virus-like" NEXT particle*) NEAR/2 vaccin*) OR nanovaccin* OR proteinvaccin* OR peptidevaccin* OR subunitvaccin* OR recombinantvaccin*):ti,ab,kw | 3056 |
| #2 | (virus or viral):*ti,ab,kw* | 54259 |
| #3 | #1 AND #2 with Cochrane Library publication date in The last 2 years, in Trials | 215 |
| Hyperlink: <https://www.cochranelibrary.com/advanced-search/search-manager?search=7822576> | | |

**Supplementary table 2: Literature search algorithms for viral vector vaccine technology landscape using MEDLINE Ovid, Embase Ovid and CENTRAL**

| **MEDLINE(R) Ovid, Date: 14.10. 2025** | | |
| --- | --- | --- |
| 1 | vector*.ti,ab,kf. | 329798 |
| 2 | (adenovir* or lentivir* or vaccinia or measles or vesicular stomatitis vir* or VSV or modified virus* or recombinant virus* or Poxvirus* or Sendai virus* or Newcastle disease virus* or Cytomegalovirus or Parainfluenza Virus 5 or Rabies virus*).ti,ab,kf. | 214908 |
| 3 | 1 or 2 | 507668 |
| 4 | Vaccines/ or exp Viral Vaccines/ | 188776 |
| 5 | vaccine*.ti,ab,kf. | 368155 |
| 6 | 4 or 5 | 415188 |
| 7 | exp Virus Diseases/ or exp Viruses/ | 1781627 |
| 8 | (virus* or viral or poxvirus or cytomegalovirus).ti,ab,kf. | 1190402 |
| 9 | 7 or 8 | 2101297 |
| 10 | 3 and 6 and 9 | 37828 |
| 11 | limit 10 to last 2 years | 5095 |
| 12 | limit 11 to (case reports or meta analysis or "review" or "scoping review" or "systematic review") | 1126 |
| 13 | 11 not 12 | 3969 |
| 14 | limit 13 to (clinical trial, all or clinical trial, phase i or clinical trial, phase ii or clinical trial, phase iii or clinical trial, phase iv or clinical trial, veterinary or clinical trial protocol or clinical trial or controlled clinical trial or human challenge trials or randomized controlled trial or randomized controlled trial, veterinary) | 157 |
| 15 | (rct or ((random* or clinical or preclinical or multicenter) adj3 (study or studies or trial*)) or ((animal* or human* or invitro or in-vitro or in vitro) adj3 (model* or challenge))).ti,ab,kf. | 1944785 |
| 16 | 13 and 15 | 477 |
| **17** | **14 or 16** | **520** |
| Hyperlink: <https://ovidsp.ovid.com/ovidweb.cgi?T=JS&NEWS=N&PAGE=main&SHAREDSEARCHID=1YwavNSmsaVXYbs5ZfPsMvr6i84Dugn2WW1XXKOhdK2zouAX0MGIV0IXzynmUJFKz> | | |
| **Embase Ovid, Date: 14.10. 2025** | | |
| 1 | exp virus vector/ | 119330 |
| 2 | vector*.ti,ab,kf. | 399891 |
| 3 | (adenovir* or lentivir* or vaccinia or measles or vesicular stomatitis vir* or VSV or modified virus* or recombinant virus* or Poxvirus* or Sendai virus* or Newcastle disease virus* or Cytomegalovirus or Parainfluenza Virus 5 or Rabies virus*).ti,ab,kf. | 271848 |
| 4 | 1 or 2 or 3 | 659330 |
| 5 | vaccine/ or exp virus vaccine/ or exp vector vaccine/ | 328127 |
| 6 | vaccine*.ti,ab,kf. | 446527 |
| 7 | 5 or 6 | 539367 |
| 8 | exp virus infection/ or exp virus/ | 2966047 |
| 9 | (virus* or viral or poxvirus or cytomegalovirus).ti,ab,kf. | 1422582 |
| 10 | 8 or 9 | 3184335 |
| 11 | 4 and 7 and 10 | 59404 |
| 12 | limit 11 to last 2 years | 10891 |
| 13 | limit 12 to (books or chapter or "review") | 1543 |
| 14 | limit 12 to (meta analysis or "systematic review") | 465 |
| 15 | 12 not (13 or 14) | 9161 |
| 16 | limit 15 to (clinical trial or randomized controlled trial or controlled clinical trial or multicenter study or phase 1 clinical trial or phase 2 clinical trial or phase 3 clinical trial or phase 4 clinical trial) | 855 |
| 17 | (rct or ((random* or clinical or preclinical or multicenter) adj3 (study or studies or trial*)) or ((animal* or human* or invitro or in-vitro or in vitro) adj3 (model* or challenge))).ti,ab,kf. | 2960680 |
| 18 | 15 and 17 | 1083 |
| **19** | **16 or 18** | **1464** |
| Hyperlink: <https://ovidsp.ovid.com/ovidweb.cgi?T=JS&NEWS=N&PAGE=main&SHAREDSEARCHID=1cBZHJ2RnT6r3nc5zYcLOhLmVPaQBbzNhMRkPkKNvlsAlrbxenro2IgPrf6YUOB0g> | | |
| **CENTRAL, Date: 14.10.2025** | | |
| #1 | (vector*):ti,ab,kw | 4336 |
| #2 | (adenovir* or lentivir* or vaccinia or measles or "vesicular stomatitis" NEXT vir* or VSV or modified NEXT virus* or recombinant NEXT virus* or Poxvirus* or Sendai NEXT virus* or "Newcastle disease" NEXT virus* or Cytomegalovirus or "Parainfluenza Virus 5" or Rabies NEXT virus*):ti,ab,kw | 6187 |
| #3 | #1 OR #2 with Cochrane Library publication date in The last 2 years, in Trials | 1096 |
| #4 | (vaccine*):ti,ab,kw | 30907 |
| #5 | (virus or viral or poxvirus or cytomegalovirus):ti,ab,kw | 55754 |
| **#6** | **#3 AND #4 AND #5 with Cochrane Library publication date in The last 2 years, in Trials** | **193** |
| Hyperlink: <https://www.cochranelibrary.com/advanced-search/search-manager?search=7826238> | | |

**Supplementary table 3: Literature search algorithms for Lassa and Nipah virus landscape using MEDLINE Ovid, Embase Ovid and CENTRAL**

| **MEDLINE(R) Ovid,** Date: 30.09.2025 | | |
| --- | --- | --- |
| 1 | exp Vaccines/ or exp Vaccines, Synthetic/ or exp Vaccination/ | 336250 |
| 2 | (vaccin* or immuniz* or immunis*).ti,ab,kf. | 565393 |
| 3 | 1 or 2 | 621461 |
| 4 | Lassa Fever/ or Lassa virus/ or Henipavirus/ or Nipah Virus/ | 2291 |
| 5 | (lassa* or nipah* or henipa*).ti,ab,kf. | 4009 |
| 6 | 4 or 5 | 4175 |
| 7 | 3 and 6 | 953 |
| 8 | (rct or ((random* or clinical or preclinical or multicenter) adj3 (study or studies or trial*)) or ((animal* or human* or invitro or in-vitro or in vitro) adj3 (model* or challenge))).ti,ab,kf. | 1939482 |
| 9 | 7 and 8 | 206 |
| 10 | limit 7 to (adaptive clinical trial or clinical study or clinical trial, all or clinical trial, phase i or clinical trial, phase ii or clinical trial, phase iii or clinical trial, phase iv or clinical trial, veterinary or clinical trial protocol or clinical trial or controlled clinical trial or equivalence trial or human challenge trials or pragmatic clinical trial or randomized controlled trial or randomized controlled trial, veterinary) | 4 |
| 11 | 9 or 10 | 206 |
| **12** | **limit 11 to last 2 years** | **61** |
| Hyperlink: <https://ovidsp.ovid.com/ovidweb.cgi?T=JS&NEWS=N&PAGE=main&SHAREDSEARCHID=6qXM1MQu49d4hqQdjAfoVq2Cr9XsLktl4MhhxXYWyhiOAkhEgTr5u1oncm3dyzz24> | | |
| **Embase Ovid,** Date: 30.09.2025 | | |
| 1 | exp vaccine/ or exp vaccination/ | 582079 |
| 2 | (vaccin* or immuniz* or immunis*).ti,ab,kf. | 672249 |
| 3 | 1 or 2 | 811813 |
| 4 | Lassa fever/ or Lassa virus/ or Henipavirus/ or Nipah virus/ | 4508 |
| 5 | (lassa* or nipah* or henipa*).ti,ab,kf. | 4687 |
| 6 | 4 or 5 | 5715 |
| 7 | 3 and 6 | 1478 |
| 8 | (rct or ((random* or clinical or preclinical or multicenter) adj3 (study or studies or trial*)) or ((animal* or human* or invitro or in-vitro or in vitro) adj3 (model* or challenge))).ti,ab,kf. | 2949640 |
| 9 | 7 and 8 | 279 |
| 10 | limit 7 to (clinical trial or randomized controlled trial or controlled clinical trial or multicenter study or phase 1 clinical trial or phase 2 clinical trial or phase 3 clinical trial or phase 4 clinical trial) | 56 |
| 11 | 9 or 10 | 307 |
| **12** | **limit 11 to last 2 years** | **93** |
| Hyperlink: <https://ovidsp.ovid.com/ovidweb.cgi?T=JS&NEWS=N&PAGE=main&SHAREDSEARCHID=26H2QJFbkzSkU6KiZEwGbZJOWewU7F9guh9iQbdoV148uf2UNqOl3HZsuJb8RL5iJ> | | |
| **CENTRAL**, Date: 30.09.2025 | | |
| ID | Search | Hits |
| #1 | (vaccin* OR immuniz* OR immunis*):ti,ab,kw | 36878 |
| #2 | (lassa* OR nipah* OR henipa*):ti,ab,kw | 63 |
| **#3** | **#1 AND #2 with Cochrane Library publication date in The last 2 years, in Trials** | **9** |
| Hyperlink: <https://www.cochranelibrary.com/advanced-search/search-manager?search=7817100> | | |
